# Human development III: Bridging Brain-Mind and Body-Mind. Introduction to “Deep” (Fractal, Poly-Ray) Cosmology

**DOI:** 10.1100/tsw.2006.157

**Published:** 2006-07-06

**Authors:** Søren Ventegodt, Tyge Dahl Hermansen, Erik Rald, Trine Flensborg-Madsen, Maj Lyck Nielsen, Birgitte Clausen, Joav Merrick

**Affiliations:** ^1^ Quality of Life Research Center, Teglgårdstræde 4-8, DK-1452 Copenhagen K, Denmark; ^2^ Research Clinic for Holistic Medicine, Copenhagen, Denmark; ^3^ Nordic School of Holistic Medicine, Copenhagen, Denmark; ^4^ Scandinavian Foundation for Holistic Medicine, Sandvika, Norway; ^5^ Interuniversity College, Graz, Austria; ^6^ Vejlby Lokalcenter, Vejlby, Denmark; ^7^ National Institute of Child Health and Human Development, Faculty of Health Sciences, Ben Gurion University, Beer-Sheva, Israel; ^8^ Center for Disability and Human Development, Faculty of Health Sciences, Ben Gurion University, Beer-Sheva, Israel; ^9^ Office of the Medical Director, Division for Mental Retardation, Ministry of Social Affairs, Jerusalem, Israel

**Keywords:** quality of life, QOL, holistic biology, clinical holistic medicine, public health, mental, emotional, cosmology, Denmark

## Abstract

Reality can be interpreted in many ways, but two distinctly different ways are the mental and the emotional interpretation. The traditional way of thinking in science today is the first: an often simple and mechanical interpretation of reality that empowers us to handle the outer physical world with great, often brutal efficiency. The development of a mind that enables us to handle the outer physical world and survive makes a lot of sense from an evolutionary perspective; the problem is that the mental reason and linear logic reduces all phenomena to well-defined interacting objects, which might not exist from a deeper perspective of reality. A more intuitive way to interpret the world makes much more sense, when it comes to our human relations. So to function as a human being, we need both these two ways of seeing the world, and two different modi operandi. In many patients, we find an internalized conflict between logical and mental reasoning on one hand, and emotional and sexual approach to reality and human needs on the other. We speculate that this conflict causes the deep emotional problems that really are the basis of most human diseases. Only by merging brain-mind and body-mind will we be whole and free and truly ourselves. We need to develop our mental understanding, deepen our cosmology, and develop our sexuality and body-mind in order to make them meet and merge. To facilitate this existential healing, we propose a third integrative way of looking at our human nature, which we call “the energetic-informational interpretation of reality”. What it does is allows us to look at both brain-mind and body-mind as a highly structured field of “energy and information”. Energy and information are actually the same from a scientific point of view; when the world is seen through the body-mind, it looks more like energy; when seen though the brain-mind, it looks more like information.

## INTRODUCTION

Science is about describing and interpreting reality. An interpretation of reality is a brain representation of reality, which carries a specific structure. Reality can be "broken down" and interpreted in several possible ways. Usually, before we develop our understanding of life and consciousness, we are able to interpret reality and its phenomena in at least one of two very different ways: a rational/mental or an emotional/intuitive way. The rational/mental way is often a mechanistic way of looking at nature, as chemistry and logical patterns of behavior, while the emotional/intuitive way is a more naive and immediate interpretation of reality with offspring in our bodily experiences. Often, we are able to use the first understanding of the world when we work, and the second understanding when we are most private, like taking care of our children or while we are making love to our partner.

The rational interpretation of the world is seen as more "clever" and more discriminative and more analytically intelligent than the intuitive interpretation, which on its own is more "synthesizing" and emotionally intelligent[1], making us see meaning in things and feel love and wholeness. When the rational and mental interpretation of reality has been accepted by the biological and the medical sciences, as it has happened with molecular biology and biomedicine, this will lead to many anomalies and paradoxes and leave many important phenomena incomprehensible.

The human consciousness is maybe the most difficult thing to explain with a mechanical approach, as the "light" of consciousness has a coherent, unifying, and global quality, which seemingly cannot be matched by any known natural, physical law in contemporary natural science, even when tried with the most brilliant mental intelligence.

When we look at the world through our feelings, we see relations and connections with no place in the rational, mental, and formal view of the world. It seems that the emotional interpretation of reality in many ways is closer to human biology than the mental interpretation[1]. In this series of papers on human development, we propose a third way of looking at nature that integrates these two perspectives. We call this third perspective the "energetic-informational interpretation of reality".

The search for an integrated perspective is really the search for a philosophy of life that integrates the mental and the emotional-physical side of man. This interesting search is a door to a more general understanding of the many ways the world can be understood: the varieties of cosmologies that exist now. They have been developed through the times by the many human civilizations on many continents, from the African Sangomas to the Australian aboriginals, Asian Indian sages, and American Maya Indians. Most interestingly, medicine seems to be one of the primary purposes for developing the cosmologies; the most complex of cosmologies, the cosmologies with the greatest concepts and most profound mystical depth, are often held by the shamans and the medicine men for the purpose of healing the people. This is not a coincidence.

Bridging the brain-mind and the body-mind is to make man whole; it is a way to heal both mental and physical diseases. The integration is not easy because it takes a deep philosophical understanding of life and self to mobilize all the hidden resources in man[2]. The new integrative interpretation of the world is what allows us to heal and even sometimes to transform completely and go into the process of radical reorganization of the whole existence — the very intense and rapid process of spontaneous self-healing we call "adult human metamorphosis". The understanding of the background of this pattern of healing is what motivates us for the quest and, interestingly, the quest to understand life better was what motivated and gave one of the authors (SV) the faith to follow the first patients through the dramatic, intense, and often very painful process of human metamorphosis 7 years ago, when it looked like a common psychosis, just with more intensity from the deep layer of existence that Carl Gustav Jung (1876–1961) called the "collective consciousness"[3]. To make the reader understand and believe what we have seen and found though the last 20 years of research in the connection between health and human consciousness, we will introduce the reader to the different aspects of a new holistic view of the world and its major phenomena: matter, life, and consciousness. This interdisciplinary understanding of human development seems to be what is needed to explain healing.

## HOLISTIC BIOLOGICAL PARADIGM

When we approach the world though the mind, we interpret it. We cannot interpret the world without a personal purpose in life to source our values and intentions. In order to make sense out of chaos, we need to be a part of the world. This makes all science highly biased, which also is the point of many philosophers of science (Popper, Kuhn, Chalmers, Fireaben, Gøtzche). In a way, it is also nice because it empowers every one of us to look for our own truth.

We have been experimenting with different interpretations of reality, as they rose when we looked at the world though different "glasses": through the brain, the world looks one way, and through emotions and intuition, the world looks quite different. A mental perspective makes it possible to analyze the world into different pieces. An emotional perspective empowers us to see the relations between people and things. To train the ability to see the world in different ways is an exciting experiment that gives us respect for the process of interpretation. Every person has his own way of looking at and understanding the world, and is simultaneously able to relate to a set of different ways to look at the world because our human organism embodies a set of faculties of intelligences that make this possible.

This modern view makes science complicated. For what can be absolutely truth, if we are so subjective and our worldview so easily manipulated? We believe that when we have understood mind and emotions well, a new set of eyes opens, allowing us to see the world in a deeper and more objective way. This might be as illusory as everything else of course, but we invite you to follow us on this journey, and we believe that in doing so, you will share our experience of a deeper understanding of life. We have called this deeper perspective *the energetic-informational interpretation of reality* or simply *the holistic paradigm,* as it brings us closer to the wholeness of the world and its phenomena than do both the mental and the emotional interpretations of the world. When we look at biology in this way, it seems possible to obtain a deeper understanding of life and human development, in the to papers that will follow.

## REFLECTIONS ON SCIENCE

Scientists often suppose that they reach their conclusions by use of mental reasoning, and scientists will normally try to avoid bias from emotions, intentions, and beliefs. Mental reasoning is the ability to analyze and group phenomena, built on logic and set theory of mathematics. Classically, science is performed by dividing reality into its parts and then describing the characteristics of these parts and their interactions. The parts can normally be subdivided again and again. By and large, the different scientific disciplines are defined by the different sizes of their study objects (see Fig. 1) and, to some extent after, if the phenomenon is subjective or objective (compare: Wilber's four quadrants[4]). Between the disciplines, strong demarcation lines exist, new regularities between interactions of units emerging at each level, respectively; this is based on the idea that reality consists of levels and each level has its own sphere with its own class of phenomena and associated laws.

Mental reason reduces all phenomena to well-defined interacting objects. We become terribly frustrated when a description (for example, of the electron as a well-defined, isolated independent particle) is not valid. Then, we try to comfort ourselves with the fact that the description of nature as particles at least persists regarding bigger things than particles, even when science shows that the quantum mechanical laws also rules for big molecules[5]. But most unfortunately, we cannot understand biological organisms by reducing them to "things". To understand why this is so, we must accept that an interpretation of reality always happens to serve a specific purpose.

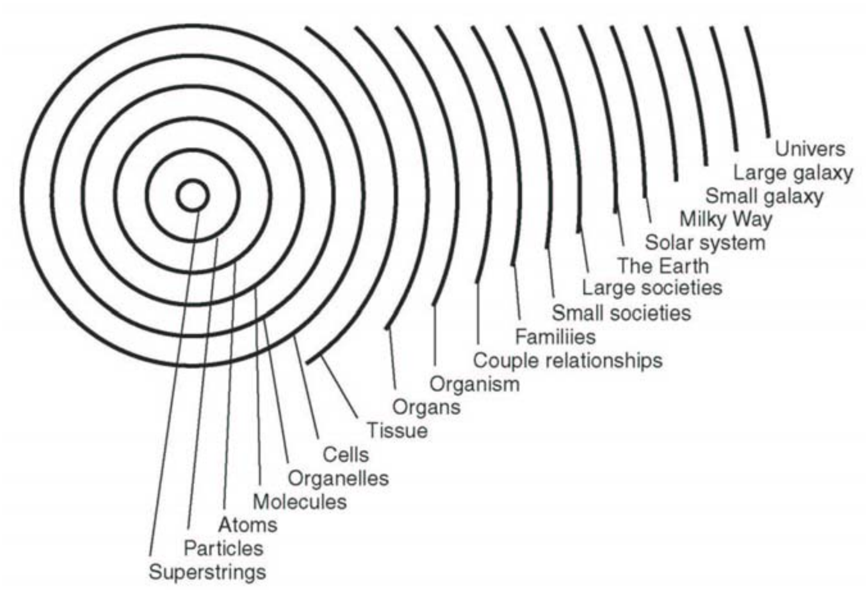

**FIGURE 1.** How reason has divided reality into scientific disciplines according to the size of the objects of investigation.

A simple and mechanical (gr. mechane = tool) interpretation of reality empowers us as human beings to handle cleverly the outer physical world through the use of tools and technologies. The ability of our reason to reduce reality to interacting elements is reflected deep down in the roots of our language, demonstrating that the brain and language have been busy with this kind of survival for many generations.

The development of a mind matching the outer world can be seen from an evolutionary perspective. The complicated use of tools and language makes us as human beings fit for survival, which is exactly what sets us apart from the apes. When we became human and developed logical reasoning, in addition to the feelings and emotional reasoning we already had as apes, we were forced into a conflict between emotions and logical reason. As apes, we had conflicts between reality and simple biological needs. The modern man developed a brain that allowed him to a far higher degree to comply with the physical world; however, the conflict between the biological needs and the outer world persists, but today the conflict has partly moved inwards in the organism and has became a conflict between the brain and the rest of the biological wholeness of man. Man's excellent ability to adapt has become his heel of Achilles.

We assume that this internalized conflict between logical and mental reason and emotional and sexual needs is the basis of all illnesses in the human being, both concerning mental diseases like schizophrenia (only known in man) and the largest fraction of the somatic illnesses (also found much more frequently in man than in apes and other animals). Man is generally very vulnerable compared to animals; brain surgery may be carried out without problems in rats under nonsterile conditions; in man this can hardly be done.

The project of science is to explore reality by means of study and experiments. However, experiments always have to be constructed, and the results always have to be interpreted. The rationalists assume that the road to truth goes through the interpretation of experimental experiences, while the emotions constitute an "irrational element", diverting reason from truth. But reason betrays, too. Reason notices most easily phenomena that make good sense. This inevitably leads to confirmation of previously established ideas and expectations. Logic is able to provide consistency in a logical system, but every logical system rests upon axioms, which we get from our intuitive understanding of the world. Reason, therefore, is in its essence nurtured by the tacit, emotional dimensions of existence, not the other way around. When discriminative reason analyzes reality into parts, it requires a "synthetic" function of emotion to group them into a meaningful synthesis. Meaning is supplied by emotionality and intuition.

Emotions betray, because a great amount of conditioned learning (learning that serves adaptation to reality at a given point in time normally motivated by survival) lies between the individual and a direct experience of reality. We have a strong tendency to mistake past for present because we do not update our adaptation to our shifting reality. We feel what we have learned to feel instead of what we really feel. Reason betrays, because it has a great tendency to notice what gives immediate meaning, which is a phenomena in accordance with previously learned fundamentals and axioms. It is self-affirmative. We are slaves of our learning and "betrayed" by both our emotions and our reason.

In order to solve these problems and obtain true knowledge, we must handle the delusion of reason as well as the delusion of emotion. Reason must be expanded through a constant attempt to introduce more roomy axioms, and to handle these in a noncontradictory way. The reality of the emotions must be cleansed of conditioned learning in order to make us experience reality without the influence of pollution from past learning. The ideal is a complete unification of emotions and reason, corresponding to man's greatest potential of knowledge. The more we cultivate our mind, the better we represent a matter in our consciousness and mental understanding. We call this the double cultivation of emotions and reason, and the application of their synthesis in the interpretation of reality, we call the interpretation paradigm.

## The Mental-Rational Interpretation of Reality

When we want to handle the outer world, we use logic, set up theories, and interpret the phenomena of the world as things, e.g., bodies or particles. The particles move in space and when they move, time is created. Their interactions reveal forces that may cause the bodies to accelerate. The resistance to acceleration corresponds to their mass. However, the forces and the masses, as well as the particles themselves, are not explained by this description.

Modern physics, including the theories of relativity and quantum mechanics, has shown that a simple, mechanical interpretation of reality, such as Newton's, is not only insufficient, but also incorrect. We have to admit that our rational interpretation of reality is in fact a simplification of reality. Relativity theory shows that gravitational forces between particles equivalently can be described as curvature in a four-dimensional space-time[6]. Space and time has to be broken down in another way. The notion of curved space-time does not supply a real understanding, but indicates that a completely new interpretation of reality is necessary for a deeper understanding of reality.

Quantum mechanics describes the electron as sometimes a wave[4,7] and sometimes a particle. This is the famous Copenhagen interpretation of quantum mechanics by Niels Henrik David Bohr (1885–1962). This interpretation violates our straightforward impression of a particle as something well defined in space. Both relativity theory and quantum mechanics points to the fact that a new interpretation of reality is needed to arrive at a proper understanding.

## The Emotional-Intuitive Interpretation of Reality

In our daily lives, we usually interpret reality with a starting point in ourselves, introspectively in our inner world, and in the outer world by looking at living organisms – above all people we love. Our attention is drawn to fluidly changing totalities. We think by association and in pictures. There are no fixed boundaries between the world and us, and there are no fixed boundaries between the parts of the world. There are in fact no parts, only totalities moving to create meaningful patterns. In the rational interpretation of reality, delimited elements are a necessary condition for interpretation, but in the emotional interpretation, we find significance and system states instead. Focus is at the patterns of rays extending throughout the levels (see Fig. 2). The rational interpretation is fixed, local, and logical, and it thinks in terms of space, time, and mass. The emotional interpretation is fluid, nonlocal, nonlogical, and not involved with concepts of space, time, and mass. It is the reality of the unconscious, of feelings and urges, and of intention and love.

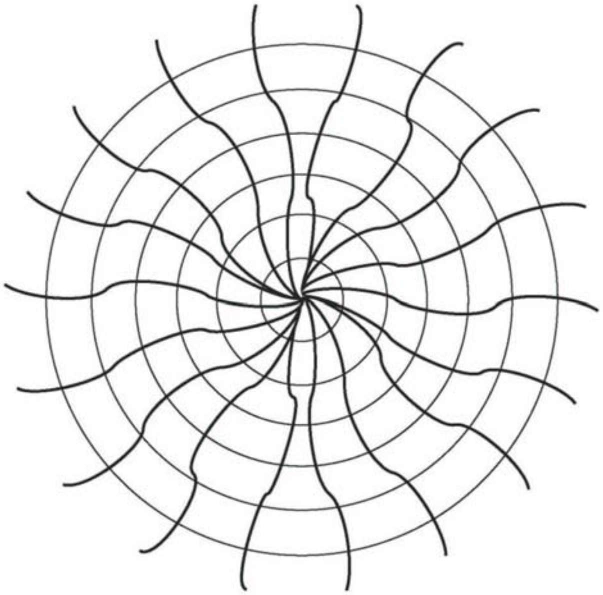

**FIGURE 2.** The aspects illustrated across the organizational levels.

## The Energetic-Informational Interpretation of Reality

In human life, the rational and the emotional interpretations of reality often conflict or seem to mutually exclude each other. The conflict between power and love is a classical conflict, well known to many people in their own life. In philosophy, the incommensurability of the body and the soul is a manifestation of the same conflict. In order to obtain true knowledge, we have to handle these discrepancies and create a synthesis of the two interpretations. We have to give solidity to the fluid emotions, and we have to make firm reason more liquid. In other words, we have to exchange the axioms of reason with axioms that can incorporate our emotional reality. Hereby the formerly disparate rational and emotional interpretations become transformed to a deeper interpretation cleansed of all the conditionings that oppose each other in the two separate interpretations.

Energy, organization, and information seem to be sufficiently elastic concepts of the simultaneous description of both the rational and the emotional interpretation of reality. The concepts of space, time, particle, and mass, therefore, have to be exchanged with the concepts of information, organization, and energy. The particles of physics lose their particle status and have to be described as patterns of structure and information containing energy. What we, according to the rational interpretation, would call a particle in space and time is, in the energetic-informational interpretation, a pattern of structure and information that can never be separated from the totality of which it is a part.

The theory of superstrings[8] has done admirable work by expanding the principle of equivalence to all basic forces of nature. But the multidimensional nature of the universe contradicts our common experience of space as three-dimensional. How can space-time have 10 or more dimensions? When mathematical formality requires extra dimensions, these are simply given status as reality. The superstrings are conceived as being very small and are able to form particles. The concept of particles belongs to the rational interpretation of reality and should be abandoned in the energetic-informational interpretation of reality. Even strings seem to be too mechanical to fit in. It is still a simplistic model of reality. The energetic-informational interpretation of reality must employ a much more complex breakdown of reality. We must seek for the complex and coherent structure of the universe to really understand life and consciousness. This is the challenge we face in the present series of papers on holistic biology.

## DISCUSSION

We believe that the internalized conflict between logical and mental reason and emotional and sexual needs is the basis of all human illnesses. The severe incongruence of the brain-mind and the body-mind destroys coherence[9,10,11]. We conclude that in order to solve these problems and obtain true knowledge, we must handle the delusion of reason as well as the delusion of emotion. Reason can be expanded through a constant attempt to introduce more spacious axioms, and to handle these in a noncontradictory way. This is the evolution of reason. A more radical way is to allow chaos and confusion and let a completely new matter of understanding appear – this is the revolution of mind that lead to deeper cosmology[12]. The more we cultivate our mind, the better we represent the world in our consciousness and mental understanding. We call this the double cultivation of emotions and reason, and the application of their synthesis in the interpretation of reality, the integrative, or "deep", cosmology.

In the concept of the rational interpretation of reality, the relativistic notion of curved space-time and the uncertainties and nonlocality of quantum mechanics indicate that a completely new interpretation of reality is necessary for a deeper understanding of reality. Both relativity theory and quantum mechanics point to the fact that what appears for the eye is not so; a new, more complex interpretation of reality is needed for a proper understanding of the world. Interestingly, both of them are irresponsible in a way; the overintellectual escapes the painful feelings and the overemotional escapes the narrowing bands from clear responsible thinking.

A true, trustworthy, mature, and "deep" cosmology will deal with all phenomena of the world in a orderly way, giving a graduated spectrum of phenomena from the dense material to the almost nonexisting spiritual[13]. In human life, the rational and the emotional interpretations of reality often conflict or seem to mutually exclude each other. In order to obtain true knowledge as well as mental, physical, social, and spiritual health, we need to handle these discrepancies and create a synthesis of the two interpretations, leading to an interpretation of reality based on concepts of energy and information. In this, the physical particles lose their particle status and feelings lose their personal and historical significance. The world is described as patterns of more or less materialized information, and more or less free energy.

What we would call "a particle in space and time" in the rational interpretation of the world and what we would call "a subjective experience" in the emotional/intuitive interpretation will, in the deep cosmology (in the energetic-informational interpretation of the world), be seen as a pattern of structure and information that can never be separated from the totality of which it is a part.

## CONCLUSION

We become terribly frustrated when a description (for example, of the electron as a well-defined, isolated particle) is not valid. Then, we try to comfort ourselves with the fact that the description of nature as particles persists, at least regarding bigger things than particles, even when science clearly demonstrates that also much larger things than small molecules, atoms and particles are ruled by the strange laws of quantum mechanics. Recently quantum interference experiments by Olaf Nairz, Markus Arndt, and Anton Zeilingerb with very large molecules have been documenting this quite convincingly[14]. But most unfortunately, we cannot understand biological organisms by reducing them to "things". To understand why this is so, we must accept that an interpretation of reality always happens to serve a specific purpose.

We assume that the internalized conflict between logical and mental reason and emotional and sexual needs is the basis of all illnesses in the human being, both concerning mental illnesses like schizophrenia (only known in man) and the largest fraction of the somatic illnesses (also found much more frequently in man than in apes and other animals).

Emotions betray because a great amount of conditioned learning exists between the individual and a direct experience of reality. Also, reason betrays because it has a great tendency to notice what gives immediate meaning, which is a phenomena in accordance with previously learned fundamentals and axioms.

In order to solve these problems and obtain true knowledge, we must handle the delusion of reason as well as the delusion of emotion. Reason must be expanded through a constant attempt to introduce more spacious axioms, and to handle these in a noncontradictory way. The reality of the emotions must be cleansed of conditioned learning in order to make us experience reality without the influence of pollution from past learning. The ideal is a complete unification of emotions and reason, corresponding to man's greatest potential of knowledge. The more we cultivate our mind, the better we represent a matter in our consciousness and mental understanding. We call this the double cultivation of emotions and reason, and the application of their synthesis in the interpretation of reality, we call the interpretation paradigm.

The rational interpretation is fixed, local, and logical, and it thinks in terms of space, time, and mass. The emotional interpretation is fluid, nonlocal, nonlogical, and not involved with concepts of space, time, and mass. It is the reality of the unconscious, of feelings and urges, and of intention and love. The energetic-informational interpretation of reality (the holistic paradigm) must employ a much more complex breakdown of reality. We must seek for the complex and coherent structure of the universe to really understand life and consciousness.

## ACKNOWLEDGMENTS

These studies were supported by grants from IMK Almene Fond. Our research in quality of life has been approved by the Copenhagen Scientific Ethical Committee under number (KF)V.100.2123/91.

## BIOSKETCHES

**Søren Ventegodt, MD, MMedSci,** is the director of the Nordic School of Holistic Health and Quality of Life Research Center in Copenhagen, Denmark. He is also responsible for a Clinical Research Clinic for Holistic Medicine in Copenhagen and is a popular speaker throughout Scandinavia. He has published numerous scientific or popular articles and a number of books on holistic medicine, quality of life, and quality of working life. His most important scientific contributions are the comprehensive SEQOL questionnaire, the very short QOL5 questionnaire, the integrated QOL theory, the holistic process theory, the life mission theory, and the ongoing Danish Quality of Life Research Survey, 1991–94 in cooperation with the University Hospital of Copenhagen and the late professor of pediatrics, Bengt Zachau-Christiansen, MD, PhD. E-mail: ventegodt@livskvalitet.org. Website: www.livskvalitet.org

**Tyge Dahl Hermansen**, **MSc** in biology, is a research assistant at the Quality of Life Research Center, Copenhagen. Research areas include molecular biology, physiology, and life quality; experimental experience in fruit flies, yeast, bacteria, and cnidarians; theoretical research in human life quality; technical knowledge in the areas of animal physiology, molecular biology, biotechnology, biochemistry, cell biology, and immunology. Publications appear in popular scientific as well as international journals. E-mail: TD@livskvalitet.org

**Erik Rald, MSc** in biology, is a research assistant at the Quality of Life Research Center, Copenhagen. His research areas and publication topics include systematics of acalypterate Diptera; history and epistemology of ecology, evolution, and taxonomy; nature preservation in nature parks; systematics, ecology, and chemistry of agaric Fungi; conservation of endangered species; assessment of nature quality in grasslands, woodlands, and urban areas. E-mail: erik_rald@scientist.com Website: http://www.dknatur.dk

**Trine Flensborg-Madsen**, **BSc, MSc** (Public Health Science) is currently a research assistant at the Institute of Preventive Medicine, Faculty of Health Sciences, University of Copenhagen, where she is finishing her PhD. Earlier, she was a research assistant at the Quality of Life Research Center in Copenhagen, where she participated in several research projects concerned with holistic health, sense of coherence, and research and follow-up of the Copenhagen Birth Cohort 1959–61. E-mail: tf@imp.hosp.dk Website: http://www.ipm.hosp.dk/person/tfm.htm

**Maj Lyck Nielsen,** Occupational Therapist, specialized in psychiatry, Holistic Therapy assistant at the Quality of Life Research Center in Copenhagen, Denmark. Student at the Nordic School of Holistic Medicine. E-mail: mln@livskvalitet.org

**Birgitte Clausen, RN,** leader of a secluded ward for people with dementia. Dementia coordinator and consultant in Aarhus, Denmark with more than 10 years of experience in adult psychiatry and dementia. During the last 4 years, she has worked together with Søren Ventegodt on the "QOL as medicine project" and on developing existential holistic group therapy. Through her articles and lectures, she has disseminated "The New Culture of Dementia" in both Denmark and Japan. She has participated in the production of several videos to order to inspire relatives, medical staff, and volunteers to understand people with dementia. This project has been granted financial support from the Ministry of Social Affairs in Denmark. She has conducted the first Danish investigation on the effect of sufficient daylight on the human spirit, activity level, and biological rhythm. E-mail: **villahvide@mail.dk**

**Joav Merrick, MD, MMedSci, DMSc,** is professor of child health and human development affiliated with the Center for Multidisciplinary Research in Aging, Zusman Child Development Center, Division of Pediatrics and Community Health at the Ben Gurion University, Beer-Sheva, Israel; the medical director of the Division for Mental Retardation, Ministry of Social Affairs, Jerusalem; and the founder and director of the National Institute of Child Health and Human Development. Dr. Merrick has numerous publications in the field of child health and human development, rehabilitation, intellectual disability, disability, health, welfare, abuse, advocacy, quality of life, and prevention and received the Peter Sabroe Child Award for outstanding work on behalf of Danish Children in 1985 and the International LEGO-Prize ("The Children's Nobel Prize") for an extraordinary contribution towards improvement in child welfare and well being in 1987. E-mail: jmerrick@internet-zahav.net. Website: www.nichd-israel.com
